# 2192 The association of preoperative functional capacity and outcomes for head and neck cancer patients undergoing definitive surgical treatment

**DOI:** 10.1017/cts.2018.306

**Published:** 2018-11-21

**Authors:** Sampat Sindhar, Dorina Kallogjeri, Troy S. Wildes, Michael S. Avidan, Jay Piccirillo

**Affiliations:** 1 Institute of Clinical and Translational Sciences, Washington University in St. Louis; 2 School of Medicine, Washington University in St. Louis

## Abstract

OBJECTIVES/SPECIFIC AIMS: To study the role functional capacity plays in surgical outcomes for head and neck cancers. METHODS/STUDY POPULATION: In this single-institution cohort study, we combined preoperative anesthesia assessment information with oncology registry data for newly-diagnosed patients with squamous cell carcinoma of the oral cavity, pharynx, and larynx (HNSCC) treated with definitive surgery at Siteman Cancer Center from 2012 to 2016. Patient-reported exercise capacity was assessed as metabolic equivalents. Metabolic equivalents<4 was defined as poor functional capacity. The primary outcome measure was overall survival (OS). Kaplan-Meir survival analysis was used to compare the survival of patients with poor functional capacity (PFC) and patients with normal functional capacity (NFC). Cox proportional hazard regression was used to explore the independent prognostic role of functional capacity on overall survival after controlling for other factors. RESULTS/ANTICIPATED RESULTS: A total of 671 patients underwent surgical treatment for HNSCC. The average age was 62 years (range: 19–94 years). Majority of the patients were male (n=481; 72%), White race (n=589; 88%), and smokers (n=528; 79%). Of 671 patients, 22% (n=146) had PFC. Two-year OS rate in PFC patients was 70% compared with 85% in NFC patients (15% difference; 95% CI: 7%–23%). Unadjusted Cox proportional hazard analysis showed that PFC patients had 2.2 times higher risk of death (95% CI: 1.5–3.2) than NFC patients. After adjustment for age at surgery, BMI, preoperative weight loss, comorbidity score, tumor site, and TNM stage the magnitude of the association between functional capacity and OS decreased (aHR=1.3; 95% CI: 0.88–1.98). DISCUSSION/SIGNIFICANCE OF IMPACT: Poor functional capacity is associated with decreased overall survival, but the magnitude of the association, while clinically meaningful, decreases after controlling for other important patient and tumor factors. Nevertheless, we believe preoperative functional capacity status is an important patient factor to consider when discussing prognosis and attempting risk stratification. We also believe that functional capacity may be associated with 30-day unplanned readmissions and 90-day complications and are currently performing chart review to ascertain this information.

